# Ethnic variation in prevalence, self-reported barriers and outcome of cataract surgery in a rural population in southwestern China: the Yunnan minority eye study

**DOI:** 10.1186/s12889-020-09009-5

**Published:** 2020-06-09

**Authors:** Wei Shen, Qing Cun, Hua Zhong, Chen-Wei Pan, Jun Li, Qin Chen

**Affiliations:** 1 Department of Ophthalmology, the Fourth Affiliated Hospital of Kunming Medical University (the Second People’s Hospital of Yunnan Province), Yunnan Eye Institute, Key Laboratory of Yunnan Province for the Prevention and Treatment of Ophthalmology, Kunming, 650032 People’s Republic of China; 2 Department of Ophthalmology, the First Affiliated Hospital of Kunming Medical University, Kunming, China; 3 School of Public Health, Medical College of Soochow University, Suzhou, China; 4 Department of Ophthalmology, the First Affiliated Hospital of Nanjing Medical University, Nanjing, 210000 People’s Republic of China

**Keywords:** Epidemiology, Cataract surgical coverage, Visual impairment, Barrier, Multiethnic, China

## Abstract

**Background:**

As a part of the Yunnan Minority Eye Studies, the purpose of this study was to determine the prevalence, barriers and visual acuity outcomes of cataract surgery in a multiethnic adult population in rural areas of southwestern China.

**Methods:**

A population-based cross-sectional survey was conducted with participants of Bai, Yi, and Han ethnicity aged ≥50 years in Yunnan. A detailed eye examination was performed. Information on the date, setting, type, and complications of cataract surgery were recorded in the examination of cataract-operated eyes.

**Results:**

Of 6546 subjects (2133 Bai ethnicity, 2208 Yi ethnicity and 2205 Han ethnicity), the prevalence of cataract surgery was 6.0%, with 4.6% in Bai, 7.0% in Yi, and 6.4% in Han ethnicity. Cataract Surgical coverage (CSC) among those with presenting visual acuity (PVA) < 20/200 in both eyes because of cataract was 53.3%, with 52.8% in Bai, 64.4% in Yi, and 45.3% in Han ethnicity. CSC was associated with Yi ethnicity, younger age, and higher education level, while unoperated cataract was associated with Han ethnicity, older age, and illiterate. The main barrier to cataract surgery was lack of awareness and knowledge, cost, and fear. Among the 525 cataract-operated eyes, PVA and best-corrected visual acuity (BCVA) of 20/63 or better was 44.5 and 67.2%, respectively, with 48.1 and 65.9% in Bai, 47.8 and 75.4% in Yi, 39.1 and 59.9% in Han ethnicity. Han ethnicity, aphakia, earlier year of surgery, lower-level surgical hospital and illiterate were associated with postoperative visual impairment defined by PVA, while Han ethnicity, aphakia, and illiterate were associated with that defined by BCVA. The principal causes of postoperative visual impairment were retinal disorders (26.8%), posterior capsule opacification (25.1%), refractive error(22.7%), and glaucoma (9.3%).

**Conclusions:**

Han ethnicity had a lower CSC and relatively poor visual outcomes compared with ethnic minorities. Further effective effort to remove barriers and provide sight restoration is warranted.

## Background

According to the World Health Organization (WHO) report of 2010, cataract is responsible for 51% of blindness and 33% of moderate and severe visual impairment [1]. We have to sigh that there are still so many people unknown that cataract surgery is the most effective treatment for cataract. As the leading cause of blindness worldwide, cataract is and should be a primary focus for public health ophthalmology. Population-based studies in different regional, racial and ethnic groups have shown significant differences in cataract surgical uptake and visual outcomes of cataract surgery. Recent population-based studies in developing countries show that the surgical uptake continues to remain low because of barriers such as cost, fear, ageism, and lack of awareness and knowledge [2–10]. The vision recovery after cataract surgery is usually not optimistic in most developing countries, and often fails to meet the recommended standards set by WHO [11].

The cataract surgical rate (CSR, cataract operations per million populations per year) in China has increased from 370 in 2000 to 1072 in 2012, but cataract is still the leading cause of blindness in China [12]. Cataract surgical coverage (CSC) and the quality of cataract surgery are also issues that still need to be addressed [2, 10, 12–21]. With the aging of Chinese population, this situation will be even more severe [22].

Yunnan with a multiethnic population including Han ethnicity and other 25 different recognized ethnic minorities is among the ethnically most diverse regions of China. The Han, Yi, and Bai people are the dominant ethnic groups of Yunnan. As an important part of the Yunnan Minority Eye Studies (YMES), the aim of this study was to determine whether ethnicity is associated with the prevalence, barriers and visual acuity outcomes of cataract surgery in a multiethnic adult population residing in the same region in rural areas of southwestern China.

## Methods

The YMES are population-based studies in different ethnic groups including the Han ethnicity (major ethnic group) and other ethnic minorities in Yunnan province using the same study program. The detailed method of YMES, as well as the prevalence and outcome of cataract surgery in Bai people, had been reported in the early stage [2]. Now, we have completed the data collection for the other two ethnicities: Yi and Han. We will put together the data of the three ethnic groups for analysis. Same random cluster sampling strategies were adopted to select Bai, Yi and Han people aged ≥50 years residing in rural areas in Yunnan, which is located in the southwestern China (eastern longitude 99^。^58′ and northern latitude 25^。^25′).

The study was approved by the Kunming Medical University Ethics Review Board and was conducted in accordance with the tenets of the World Medical Association’s Declaration of Helsinki.

The study procedures and definition of indicators related to cataract surgery have been described in detail reported previously [2, 22–25]. The qualified subjects were invited to study at the local community facilities. Presenting visual acuity (PVA), best corrected visual acuity (BCVA) was recorded and a comprehensive eye examination was carried out by an experienced ophthalmologist. Height, weight, systolic blood pressure, diastolic blood pressure and pulse rate for all subjects are measured using standard methods. A structured questionnaire was used to obtain information based on demographic data, educational level, socioeconomic status, smoking, alcohol consumption, personal ophthalmic and systemic disease history, and medication intake. Information on the date, setting, type, and complications of cataract surgery were recorded in the examination of cataract-operated eyes. Those with unoperated cataracts were asked why they did not receive cataract surgery. The questionnaire in Chinese provided several alternative common reasons for non-operation, including “unaware of cataract”, “no need to have cataract surgery”, “fear of surgery”, “fear of further loss of vision after surgery”, “could not afford to undergo cataract surgery”, “not accompanied by others for surgery”, “being told that there was a contraindication”, “being told to wait until the cataract gets mature”, and “undetermined” [2]. Cataract was diagnosed when lens opacity was commensurate with BCVA< 20/25 and there were no other abnormalities that can explain the decrease in VA [2].

Detailed definitions of post-operative cataract presumed severe visual impairment or blindness, unoperated cataract severe visual impairment or blindness, the social burden of cataract-related visual impairment and blindness and CSC among the cataract-related severe visual impairment or blindness are as described elsewhere [2]. We presumed that the cataract-operated eyes were severe visual impairment or blindness in the study because the indications for cataract surgery adopted by many eye care facilities in rural China were cataract-induced severely visual impairment or blindness.

### Data management and analysis

Statistical analyses were performed using SPSS 20.0 for Windows (SPSS, Inc., Chicago, IL, USA). Effect estimates were calculated as odds ratio (ORs) and 95% confidence intervals (CIs). A *P* value < 0.05 levels was considered to be statistically significant.

One-way analysis of variance was used to compare continuous variables, and chi-square test was used to compare categorical variables across different ethnic groups. The prevalence of cataract surgery and CSC among the cataract-related severely visually impaired or blind population in the study population was tabulated by ethnicity, age, gender, and education. Univariate analysis were used to investigate the association of ethnicity, age, gender, education, and other factors of interest with operated cataract, post-operative cataract with presumed severe visual impairment or blindness, unoperated cataract with severe visual impairment or blindness, the social burden of cataract-related severe visual impairment and blindness, and CSC among the cataract-related severely visually impaired or blind population. In the multivariate logistic regression analyses models, only factors that were significantly different in univariate analysis (*P* < 0.10) or factors of scientific importance were retained, such as age, gender, ethnicity.

PVA and BCVA in cataract-operated eyes were listed and compared according to ethnicity, age, gender, education, intraocular lens (IOL) status, year of surgery and surgical setting. VA was classified into four categories: ≥20/32, normal vision; 20/40 to 20/63, mild visual impairment; < 20/63 to 20/200, moderate visual impairment; < 20/200, severe visual impairment or blindness. Both univariate analysis and multiple logistic regression were used to determine the association of ethnicity, age, gender, education, IOL status, year of surgery, and surgical setting with PVA and BCVA less than 20/63 in postoperative eyes. Principal causes of visual impairment or blindness in cataract-operated eyes with PVA and BCVA less than 20/63 were identified.

## Results

After excluding paticipantints with mental retardation, mental disorder or deafness, the data analysis included 6546 subjects. 2133 (77.8%) Bai ethnicity, 2208 (80.5%) Yi ethnicity and 2205 (82.0%) Han ethnicity were participated in this study, respectively [24]. The response rate of men (65.6%) is significantly lower than that of women (93.5%). Among all age groups, the 50–59 age group (67.4%) had the lowest response rate.

Table 1 shows the differences in demographic and systemic characteristics of the three ethnic groups. Adults of the Han ethnic group were less educated, oldest, had the lowest systolic and diastolic blood pressures; were most likely to smoke and suffer from diabetes. Adults of the Bai ethnic groups were best educated, youngest, heaviest, tallest, had highest systolic and diastolic blood pressures; were least likely to smoke and drink alcohol. Adults of the Yi ethnic group were lightest, shortest; were least likely to suffer from diabetes, and were most likely to smoke and drink alcohol.

**Table 1:** Demographic and systemic factors among the three ethnic groups

	Han ethnicity	Bai ethnicity	Yi ethnicity	*P* value
**Age (years)**	65.4 (9.5)	64.6 (9.0)	65.0 (9.2)	0.017
**Female gender**	1321 (59.9)	1364 (63.9)	1258 (57.0)	< 0.001
**No formal education**	1188 (53.9)	759 (35.6)	1142 (51.7)	< 0.001
**Weight (kg)**	52.1 (9.1)	54.8 (9.9)	49.2 (9.5)	< 0.001
**Height (cm)**	156.2 (8.3)	158.2 (11.5)	155.3 (8.5)	< 0.001
**Body mass index (kg/m**^**2**^**)**	21.3 (3.2)	22.0 (5.2)	20.3 (3.2)	< 0.001
**Systolic blood pressure (mmHg)**	139.8 (24.8)	145.6 (25.6)	143.0 (26.0)	< 0.001
**Diastolic blood pressure (mmHg)**	78.1 (13.3)	80.2 (14.6)	78.6 (13.9)	< 0.001
**Diabetes**	57 (2.6)	44 (2.1)	24 (1.1)	0.001
**Smoking**	583 (26.4)	502 (23.5)	583 (26.4)	< 0.001
**Alcohol intake**	454 (20.6)	284 (13.7)	593 (26.9)	< 0.001

Continuous variables are expressed as mean (standard deviation). Categorical variables are expressed as frequencies (percentages)

Table 2 depicts the prevalence of cataract surgery for each ethnic group by age, gender, and education. 395 people (525 eyes) had undergone cataract surgery in one (265 subjects, 67.1%) or both eyes (130 subjects, 32.9%), representing a cataract surgery prevalence of 6.0% (95% CI, 5.5–6.6%), age- and sex-standardized prevalence 4.9% based on the population data of Yunnan Province in the sixth census of China in 2010 [26]. Bai ethnicity (4.6%) had a lowest cataract surgery prevalence compared with those of Han (6.4%) or Yi (7.0%).

**Table 2:** Cataract surgery for each ethnic group by age, gender, and education

Variables	Han ethnicity			Bai ethnicity			Yi ethnicity			Total		
	Examined	Post-operative persons		Examined	Post-operative persons		Examined	Post-operative persons		Examined	Post-operative persons	
	No. (%)	No.	%(95% *CI*)	No. (%)	No.	%(95% *CI*)	No. (%)	No.	%(95% *CI*)	No. (%)	No.	%(95% *CI*)
**Age(years)**												
**50–59**	712 (32.3)	8	1.1 (0.4–1.9)	715 (33.5)	7	1.0 (0.3–1.7)	690 (31.3)	11	1.6 (0.7–2.5)	2117 (32.3)	26	1.2 (0.8–1.7)
**60–69**	755 (34.2)	43	5.7 (4.0–7.4)	775 (36.3)	21	2.7 (1.6–3.9)	814 (36.9)	32	3.9 (2.6–5.3)	2344 (35.8)	96	4.1 (3.3–4.9)
**70–79**	560 (25.4)	63	11.3 (8.6–13.9)	525 (24.5)	50	9.5 (7.0–12.0)	567 (25.7)	83	14.6 (11.7–17.6)	1652 (25.2)	196	11.9 (10.3–13.4)
**80+**	178 (8.1)	28	15.7 (10.4–21.1)	118 (5.5)	21	17.8 (10.0–24.7)	137 (6.2)	28	20.4 (13.7–27.2)	433 (6.6)	77	17.8 (14.1–21.4)
**Gender**												
**Male**	884 (40.1)	54	6.1 (4.5–7.7)	769 (36.1)	35	4.6 (3.1–6.0)	950 (43.0)	52	5.5 (4.0–6.9)	2603 (39.8)	141	5.4 (4.6–6.3)
**Female**	1321 (59.9)	88	6.7 (5.3–8.0)	1364 (63.9)	64	4.7 (3.6–5.8)	1258 (57.0)	102	8.1 (6.6–9.6)	3943 (60.2)	254	6.4 (5.7–7.2)
**Education (years)**												
**0**	1188 (53.9)	103	8.7 (7.1–10.3)	759 (35.6)	53	3.4 (1.8–4.9)	1142 (51.7)	112	9.8 (8.1–11.5)	3089 (47.2)	268	8.7 (7.7–9.7)
**1–6**	742 (33.7)	27	3.6 (2.3–5.0)	868 (40.7)	29	3.3 (2.2–4.5)	763 (34.6)	34	4.5 (3.0–5.9)	2373 (36.3)	90	3.8 (3.0–4.6)
**> 6**	275 (12.5)	12	4.4 (2.0–6.8)	506 (23.7)	17	7.0 (5.2–8.8)	303 (13.7)	8	2.6 (0.8–4.5)	1084 (16.6)	37	3.5 (2.4–4.6)
**Total**	2205 (100)	142	6.4 (5.4–7.5)	2133 (100)	99	4.6 (3.8–5.5)	2208 (100)	154	7.0 (5.9–8.0)	6546 (100)	395	6.0 (5.5–6.6)

*CI* Confidence Interval

In univariate analysis, cataract surgery prevalence was significantly associated with Yi and Han ethnicity (*P* = 0.003), increased age (*P* < 0.001), lower education level (*P* < 0.001), lower weight (*P* < 0.001), lower height (*P* < 0.001), lower levels of diastolic blood pressure (*P* = 0.003), diabetes history (*P* = 0.011), no smoking (*P* < 0.001), and no alcohol intake (*P* < 0.001).

Table 3 shows the multiple logistic regression which revealed that increasing age, Han and Yi ethnicity, lower educational level, diabetes history, and no alcohol intake were significantly associated with a higher prevalence of cataract surgery in this study.

**Table 3:** Multivariate analysis on the associated factors for cataract surgery

Variables	Regression coefficent	Adjusted OR (95% CI)^a^	*P* value
**Ethnicity**			
Bai vs. Han	−0.358	0.70(0.52–0.93)	0.014
Yi vs. Han	0.200	1.22 (0.96–1.56)	0.111
**Age (years)**			
Per year increase	0.096	1.10 (1.09–1.12)	< 0.001
**Gender**			
Female vs. male	0.131	1.14 (0.87–1.50)	0.355
**Education (years)**			
1–6 vs. 0	−0.310	0.73 (0.56–0.96)	0.023
> 6 vs. 0	−0.061	0.94 (0.63–1.40)	0.762
**Weight (kg)**			
Per kg increase	−0.110	0.99(0.98–1.00)	0.087
**Height (cm)**			
Per cm increase	−0.010	0.99 (0.98–1.00)	0.149
**Systolic blood pressure (mmHg)**			
Per mmHg increase	−0.003	1.00 (0.99–1.00)	0.164
**Diastolic bloodpressure (mmHg)**			
Per mmHg increase	−0.005	1.00 (0.99–1.00)	0.253
**Diabetes**			
No vs. Yes	−0.922	0.40 (0.22–0.72)	0.002
**Smoking**			
Ever vs. never	−0.020	0.98 (0.72–1.33)	0.899
**Alcohol intake**			
Ever vs. never	−0.438	0.65 (0.47–0.88)	0.006

^a^Adjusted for ethnicity, age, gender, education, diabetes history, and alcohol intake

Table 4 demonstrates postoperative and unoperated persons with cataract-related severe visual impairment or blindness (< 20/200) by ethnicity, age, gender, and education. Of the 395 postoperative persons, 276 were presumed to have been bilaterally severely visually impaired or blind (PVA < 20/200) at the time of initial surgery, including 130 bilaterally postoperative persons and 146 unilaterally postoperative persons with a severely visually impaired or blind fellow eye. Another 242 subjects were unoperated persons with cataract-related severe visual impairment or blindness, who were bilaterally severely visually impaired or blind (PVA < 20/200) because of cataract in one or both eyes. The social burden of cataract-related severe visual impairment or blindness (PVA < 20/200) included 518 (7.9%) of the study subjects. CSC among cataract-related severe visual impairment or blindness (PVA < 20/200) was 53.3%. Adults of Han ethnicity (45.3%) had a lowest CSC compared with those of Bai (52.8%) or Yi (64.4%).

**Table 4:** Postoperative and unoperated persons with cataract-related severe visual impairment or blindness by ethnicity, age, gender, and education

Variables	Examined	Cataract-operated persons				Unopreted persons		Cataract social burdon		Cataract surgical coverage (%)
		All operated		Presumed severe visual impairment or blindness^ac^		Cataract severe visual impairment or blindness^a^				
	No.	No.	Prevalence^b^	No.	Prevalence^b^	No.	Prevalence^b^	No	Prevalence^b^	
**Ethnicity**										
**Han**	2205	142	6.4	97	4.4	117	5.3	214	9.7	45.3
**Bai**	2133	99	4.6	76	3.6	68	3.2	144	6.8	52.8
**Yi**	2208	154	7.0	103	4.7	57	2.6	160	7.2	64.4
**Age(years)**										
**50–59**	2117	26	1.2	15	0.7	8	0.4	23	1.1	65.2
**60–69**	2344	96	4.1	63	2.7	36	1.5	99	4.2	63.6
**70–79**	1652	196	11.9	133	8.1	123	7.4	256	15.5	52.0
**80+**	433	77	17.8	65	15.0	75	17.3	140	32.3	46.4
**Gender**										
**Male**	2603	141	5.4	93	3.6	86	3.3	179	6.9	52.0
**Female**	3943	254	6.4	183	4.6	156	4.0	339	8.6	54.0
**Education(years)**										
**0**	3089	268	8.7	197	6.4	169	5.5	366	11.8	53.8
**1–6**	2373	90	3.8	59	2.5	65	2.7	124	5.2	47.6
**> 6**	1084	37	3.4	20	1.8	8	0.7	28	2.6	71.4
**Total**	6546	395	6.0	276	4.2	242	3.7	518	7.9	53.3

^a^Visual acuity < 20/200

^b^Prevalence per 100 examined participants

^c^Includes all bilaterally operated persons and unilaterally operated persons with a blind fellow eye

Table 5 shows the association of ethnicity, age, gender, and education with cataract-related severe visual impairment or blindness (< 20/200) by univariate analysis. Post-operative cataract with presumed severe visual impairment or blindness associated with increased age (*P* < 0.001), female gender (*P* = 0.035), and illiteracy (*P* < 0.001). Unoperated cataract with severe visual impairment or blindness associated with Han ethnicity (*P* < 0.001), increased age (*P* < 0.001) and little or no education (*P* < 0.001). The burden of cataract-related severe visual impairment or blindness was significantly associated with Han ethnicity (*P* < 0.001), increased age (*P* < 0.001), female gender (*P* = 0.012), and little or no education (*P* < 0.001). CSC was significantly associated with Yi ethnicity (*P* = 0.001) and younger age (*P* = 0.002). Table 6 further shows the results of multiple logistic regression analysis. Post-operative cataract with presumed severe visual impairment or blindness associated with increased age and illiteracy. Unoperated cataract with severe visual impairment or blindness associated with Han ethnicity, increased age and little or no education. The burden of cataract-related severe visual impairment or blindness was significantly associated with Han ethnicity, increased age, female gender, and little or no education. The lower CSC was significantly associated with Han ethnicity, increased age and less education level.

**Table 5:** Association of ethnicity, age, gender, and education with cataract-related severe visual impairment or blindness (< 20/200) by univariate analysis

Variables	Post-operative presumed severely visually impaired or blind		Unoperated cataract severely visually impaired or blind		Cataract social burdon		Surgical coverage	
	Test statistics	*P* value	Test statistics	*P* value	Test statistics	*P* value	Test statistics	*P* value
**Ethnicity**	3.536	0.171	25.306	< 0.001	15.018	< 0.001	13.365	0.001
**Age(years)**	253,654	< 0.001	351.527	< 0.001	654.945	< 0.001	10.159	0.002
**Gender**	4.431	0.035	1.875	0.171	6.372	0.012	0.193	0.660
**Education(years)**	68.404	< 0.001	60.080	< 0.001	131.436	< 0.001	5.367	0.068

**Table 6:** Association of ethnicity, age, gender, and education with cataract-related severe visual impairment or blindness (< 20/200) by multiple logistic regression analysis

Variables		Post-operative presumed severely visually impaired or blind			Unoperated cataract severely visually impaired or blind			Cataract social burdon			Surgical coverage		
		Regression coefficent	Adjusted OR (95% CI)^a^	*P* value	Regression coefficent	Adjusted OR (95% CI)^b^	*P* value	Regression coefficent	Adjusted OR (95% CI)^c^	*P* value	Regression coefficent	Adjusted OR (95% CI)^b^	*P* value
**Ethnicity**	**Bai vs. Han**	−0.043	0.96 (0.70–1.32)	0.792	−0.361	0.70 (0.51–0.96)	0.027	− 0.265	0.77 (0.60–0.97)	0.029	0.294	1.34 (0.87–2.08)	0.187
	**Yi vs. Han**	0.136	1.15 (0.86–1.53)	0.361	−0.700	0.50 (0.36–0.69)	< 0.001	− 0.263	0.77 (0.61–0.97)	0.024	0.784	2.19 (1.43–3.36)	< 0.001
**Age(years)**	**Per year increase**	0.099	1.10 (1.09–1.12)	< 0.001	0.134	1.14 (1.12–1.16)	< 0.001	0.127	1.13 (1.12–1.15)	< 0.001	=0.043	0.96 (0.93–0.99)	0.001
**Gender**	**Female vs. Male**	0.233	1.26 (0.96–1.67)	0.100	0.211	1.24 (0.92–1.67)	0.166	0.275	1.32 (1.06–1.63)	0.012	0.063	1.07 (0.71–1.60)	0.761
**Education(years)**	**1–6 vs. 0**	−0.470	0.63 (0.46–0.85)	0.003	−0.027	0.97 (0.71–1.33)	0.867	−0.170	0.84 (0.66–1.07)	0.168	−0.443	0.64 (0.41–1.00)	0.049
	**> 6 vs.0**	−0.433	0.65 (0.40–1.05)	0.080	−0.945	0.39 (0.19–0.81)	0.012	−0.456	0.63 (0.41–0.97)	0.037	0.559	1.75 (0.73–4.21)	0.212

*OR* odds ratios, *CI* Confidence Interval

^a^Adjusted for age, gender, and education

^b^Adjusted for ethnicity, age, and education

^c^Adjusted for ethnicity, age, gender, and education

Subjects with severely visually impaired or blind because of cataract at least one eye mentioned that the most common reason for not receiving cataract surgery was that they were “unaware of cataract” (215 person, 62.0%), followed by “being told to wait until the cataract gets mature” (55 persons, 15.9%), “could not afford to undergo cataract surgery” (27 persons, 7.8%), “fear of further loss of vision after surgery” and “fear of surgery” (18 persons, 5.2%), “being told that there was a contraindication” (13 person, 3.7%),“no need to have cataract surgery” (11 persons, 3.2%), “not accompanied by others for surgery” (1 person, 0.3%), and “undetermined” (7 person, 2.0%).The constituent ratio of self-report barriers to cataract surgery was somewhat different among three ethnicities.

The percentage of eyes that were pseudophakic was 86.7% of 525 cataract-operated eyes, with 92.5% in Yi, 85.3% in Han, and 79.8% in Bai ethnicity. Multivariate logistic regression models revealed that there was no statistically significant difference between ethnic groups after adjusted for the year of surgery (Bai vs. Han OR 0.955, P = 0.898; Yi vs. Han OR 1.477, P = 0.291). Of 525 cataract-operated eyes, 84% were operated after 2001, 14.3% were operated before 2000, and the others (1.7%) were uncertain. The percentage of pseudophakic after 2000 was 93.7%, compared with 50.7% during or before 2000. Both univariate analysis (*P* < 0.001) and multivariate logistic regression (OR 0.070, *P* < 0.001) were show that higher percentage of pseudophakic was significantly associated with cataract surgeries in recent years.

Table 7 demonstrates PVA and BCVA of the 525 cataract-operated eyes by ethnicity, age, gender, education, intraocular lens status, year of surgery, and surgical setting. On the whole, we found a poor vision outcome (VA < 20/63) in 55.5% of eyes with PVA and in 32.8% with BCVA. In univariate analysis, higher prevalence of postoperative visual impairment (VA < 20/63) by both PVA and BCVA was significantly associated with Han ethnicity (*P* = 0.001), older age (*P* = 0.022), female (*P* = 0.001), illiteracy (*P* < 0.001), aphakia (*P* < 0.001), and year of surgery (*P* < 0.001). In multivariate logistic regression (Table 8), Han ethnicity, illiteracy, aphakia, earlier surgery year, and lower level surgical setting was significantly associated with postoperative visual impairment by PVA, while Han ethnicity, illiteracy, aphakia was significantly associated with postoperative visual impairment by BCVA.

**Table 7:** Presenting and best-corrected visual acuity of cataract-operated eyes by ethnicity, age, gender, education, intraocular lensstatus, year of surgery, and surgical setting

Variables	NO.(%) of eyes	NO.(%) by presenting visual acuity				NO.(%) best-corrected visual acuity			
		≥20/32	20/40–20/63	< 20/63–20/200	< 20/200	≥20/32	20/40–20/63	< 20/63–20/200	< 20/200
**Ethnicity**									
**Han**	197 (37.5)	24 (12.2)	53(26.9)	60 (30.5)	60 (30.5)	63 (32.0)	55 (27.9)	34 (17.3)	45 (22.8)
**Bai**	129 (24.6)	29 (22.5)	33 (25.6)	30 (23.3)	37 (28.7)	78 (39.2)	72 (36.2)	30 (15.1)	19 (9.5)
**Yi**	199 (37.9)	27 (13.6)	68 (34.2)	72 (36.2)	32 (16.1)	55 (42.6)	30 (23.3)	14 (10.9)	30 (23.3)
**Age(years)**									
**50–59**	31 (5.9)	10 (32.3)	6 (19.4)	7 (22.6)	8 (25.8)	18 (58.1)	7 (22.6)	3 (9.7)	3 (9.7)
**60–69**	124 (23.6)	25 (20.2)	42 (33.9)	31 (25.0)	26 (21.0)	56 (45.2)	36 (29.0)	11 (8.9)	21 (16.9)
**70–79**	263 (50.1)	35 (13.3)	76 (28.9)	91 (34.6)	61 (23.2)	94 (35.7)	80 (30.4)	49 (18.6)	40 (15.2)
**80+**	107 (20.4)	10 (15.2)	30 (28.0)	33 (30.8)	34 (31.8)	28 (26.2)	34 (31.8)	15 (14.0)	30 (28.0)
**Gender**									
**Male**	193 (36.8)	43 (22.3)	62 (32.1)	54 (28.0)	34 (17.6)	88 (45.6)	60 (31.1)	21 (10.9)	24 (12.4)
**Female**	332 (63.2)	37 (11.1)	92 (27.7)	108 (32.5)	95 (28.5)	108 (32.5)	97 (29.2)	57 (17.2)	70 (21.1)
**Education(years)**									
**0**	355 (67.6)	38 (10.7)	96 (27.0)	123 (34.6)	98 (27.6)	109 (30.7)	106 (29.9)	71 (20.0)	69 (19.4)
**1–6**	124 (23.6)	23 (18.5)	45 (36.3)	33 (26.6)	23 (18.5)	56 (45.2)	43 (34.7)	6 (4.8)	19 (15.3)
**> 6**	46 (8.8)	19 (15.2)	13 (28.3)	6 (13.0)	8(17.4)	31 (67.4)	8 (17.4)	1 (2.2)	6 (13.0)
**IOL status**									
**Pseudophakic**	455 (86.7)	79 (17.4)	151 (33.2)	150 (33.0)	75 (16.5)	195 (42.9)	140 (30.8)	61 (13.4)	59 (13.0)
**Aphakic**	70 (13.3)	1 (1.4)	3 (4.3)	12 (17.1)	54 (77.1)	1 (1.4)	17 (24.3)	17 (24.3)	35 (50.0)
**Year of surgery**									
**≥ 2001**	441(84.0)	77 (17.5)	144 (32.7)	139 (31.5)	81 (18.4)	187 (42.4)	133 (30.2)	62 (14.1)	59 (13.0)
**≤ 2000**	75 (14.3)	3 (4.0)	10 (13.3)	21 (28.0)	41 (54.7)	8 (10.7)	24 (32.0)	15 (20.0)	28 (37.3)
**Missing**	9 (1.7)	0 (0)	0 (0)	2 (22.2)	7 (77.8)	1 (11.1)	0 (0)	1 (11.1)	7 (77.8)
**Surgical setting**									
**County/ township**	432 (82.3)	64 (14.8)	120 (27.8)	143 (33.1)	105 (24.3)	158 (36.6)	130 (30.1)	67 (15.5)	77 (17.8)
**Provincial/ municipal**	93 (17.7)	16 (17.2)	34 (36.6)	19 (20.4)	24 (25.8)	38 (40.9)	27 (29.0)	11 (11.8)	17 (18.3)
Total	525 (100.0)	80 (15.2)	154 (29.3)	162 (30.9)	129 (24.6)	196 (37.3)	157 (29.9)	78 (14.9)	94 (17.9)

**Table 8:** Effect of variables on presenting and best-corrected visual acuity< 20/63 in cataract-operated eyes by multiple logistic regression analysis

Variables	Presenting visual acuity			Best-corrected visual acuity		
	Regression coefficent	Adjusted OR (95% CL)^a^	*P* value	Regression coefficent	Adjusted OR (95% CL)^a^	*P* value
**Ethnicity**						
**Han**	Reference			Reference		
**Bai**	−0.584	0.56 (0.33–0.94)	0.029	−0.205	0.81 (0.48–1.38)	0.443
**Yi**	− 0.243	0.78 (0.51–1.21)	0.268	−0.631	0.53 (0.34–0.84)	0.007
**Age(years)**						
**50–59**	Reference			Reference		
**60–69**	−0.383	0.68 (0.27–1.70)	0.412	0.006	1.01 (0.34–2.96)	0.991
**70–79**	0.20	1.02 (0.43–2.45)	0.965	0.439	1.55 (0.55–4.34)	0.404
**80+**	−0.130	0.88 (0.34–2.26)	0.787	0.645	1.91 (0.65–5.61)	0.241
**Gender**						
**Male**	Reference			Reference		
**Female**	0.319	1.38 (0.89–2.12)	0.150	0.426	1.53 (0.95–2.46)	0.079
**Education(years)**						
**0**	Reference			Reference		
**1–6**	−0.728	0.48 (0.31–0.76)	0.002	−0.994	0.37 (0.22–0.63)	< 0.001
**> 6**	−1.348	0.26 (0.12–0.55)	< 0.001	−1.343	0.26 (0.11–0.63)	0.003
**IOL status**						
**Pseudophakic**	Reference			Reference		
**Aphakic**	2.470	11.82 (4.02–34.76)	< 0.001	2.056	7.82 (4.28–14.28)	< 0.001
**Year of surgery**						
**≥ 2001**	Reference			Reference		
**≤ 2000**	1.115	3.05 (1.49–6.26)	0.002	0.603	1.83 (0.98–3.40)	0.057
**Surgical setting**						
**County/ township**	Reference			Reference		
**Provincial/ municipal**	−0.581	0.56 (0.34–0.92)	0.021	−0.143	0.87 (0.51–1.47)	0.597

^a^Adjusted for all other variable in table

Table 9 reveals the principal cause of presenting and best-corrected visual acuity< 20/63 in cataract-operated eyes. The leading causes of postoperative visual impairment by PVA were retinal disorders (78/291), posterior capsule opacification (PCO) (73/291) and refractive error (66/291), while those of postoperative visual impairment by BCVA were retinal disorders (65/172), PCO (28/172) and glaucoma (25/172). The main causes of postoperative visual impairment in each ethnic group have their own characteristics. The causes of visual impairment were somewhat different between aphakic and pseudophakic eyes. Avoidable visual impairment caused by refractive error, PCO, and pterygium accounted for 50.2% of cataract-operated eyes with visual impairment, in which Yi 58.7%, Bai 46.3%, and Han 45.0%.

**Table 9:** Principal cause of presenting and best-corrected visual acuity< 20/63 in cataract-operated eyes. No. (%)

Principal cause	Presenting visual acuity						Best-corrected visual acuity					
	Ethnicity			IOL status		Total	Ethnicity			IOL status		Total
	Han	Bai	Yi	Aphakic	Pseudophakic		Han	Bai	Yi	Aphakic	Pseudophakic	
**Refractive error/uncorrected aphakia**	22 (18.3)	22 (32.8)	22 (21.2)	18 (27.3)	48 (21.3)	66 (22.7)	8 (10.1)	0 (0)	2 (4.1)	8 (15.4)	2 (1.7)	10 (5.8)
**Retinal disorder**	35 (29.2)	15 (22.4)	29 (27.1)	15 (22.7)	63 (28.0)	78 (26.8)	28 (35.4)	14 (31.8)	23 (46.9)	13 (25.0)	52 (43.4)	65 (37.8)
**Age-related macular degeneration**	20 (16.7)	8 (11.9)	16 (15.4)	6 (9.1)	38 (16.9)	44 (15.1)	14 (17.7)	8 (18.2)	12 (24.5)	6 (11.5)	28 (23.3)	34 (19.8)
**Macular epiretinal membrane**	2 (1.7)	0 (0)	1 (1.0)	0 (0)	3 (1.3)	3 (1.0)	1 (1.3)	0 (0)	1 (2.0)	0 (0)	2 (1.7)	2 (1.2)
**Macular hole**	0 (0)	1 (1.5)	0 (0)	0 (0)	1 (0.4)	1 (0.3)	0 (0)	1 (2.3)	0 (0)	0 (0)	1 (0.8)	1 (0.6)
**Retinal detachment**	3 (2.5)	1 (1.5)	3 (2.9)	2 (3.0)	5 (2.2)	7 (2.4)	3 (3.8)	1 (2.3)	3 (6.1)	2 (3.8)	5 (4.2)	7 (4.1)
**Angiogenic retinopathy**	1 (0.8)	0 (0)	0 (0)	0 (0)	1 (0.4)	1 (0.3)	1 (1.3)	0 (0)	0 (0)	0 (0)	1 (0.8)	1 (0.6)
**Diabetic retinopathy**	1 (0.8)	0 (0)	0 (0)	0 (0)	1 (0.4)	1 (0.3)	1 (1.3)	0 (0)	0 (0)	0 (0)	1 (0.8)	1 (0.6)
**Proliferative vitreoretinopathy**	0 (0)	0 (0)	2 (1.9)	2 (3.0)	0 (0)	2 (0.7)	0 (0)	0 (0)	1 (2.0)	1 (1.9)	0 (0)	1 (0.6)
**Retinitis pigmentosa**	2 (1.7)	0 (0)	1 (1.0)	0 (0)	3 (1.3)	3 (1.0)	2 (2.5)	0 (0)	1 (2.0)	0 (0)	3 (2.5)	3 (1.7)
**Vitreous haze**	0 (0)	2 (3.0)	0 (0)	2 (3.0)	0 (0)	2 (0.7)	0 (0)	2 (4.5)	0 (0)	2 (3.8)	0 (0)	2 (1.2)
**High myopic retinopathy**	6 (5.0)	3 (4.5)	6 (5.0)	3 (4.5)	11 (4.9)	14 (4.8)	6 (7.6)	2 (4.5)	5 (10.2)	2 (3.8)	11 (9.2)	13 (7.6)
**Posterior capsule opacification**	30 (25.0)	5 (7.5)	38 (36.5)	4 (6.1)	69 (30.7)	73 (25.1)	12 (15.2)	5 (11.4)	11 (22.4)	3 (5.8)	25 (20.8)	28 (16.3)
**Glaucoma**	12 (10.0)	10 (14.9)	5 (4.8)	8 (12.1)	19 (8.4)	27 (9.3)	11 (13.9)	10 (22.7)	4 (8.2)	8 (15.4)	17 (14.2)	25 (14.5)
**Optic atrophy**	10 (8.3)	8 (11.9)	5 (4.8)	13 (19.7)	10 (4.4)	23 (7.9)	9 (11.4)	8 (18.2)	4 (8.2)	12 (23.1)	9 (7.5)	21 (12.2)
**Corneal opacity/scar**	5 (4.2)	1 (1.5)	3 (2.9)	4 (6.1)	5 (2.2)	9 (3.1)	5 (6.3)	1 (2.3)	3 (6.1)	4 (7.7)	5 (4.2)	9 (5.2)
**Pterygium**	2 (1.7)	4 (6.0)	1 (1.0)	2 (3.0)	5 (2.2)	7 (2.4)	2 (2.5)	4 (9.1)	0 (0)	2 (3.8)	4 (3.3)	6 (3.5)
**Uveitis**	2 (1.7)	0 (0)	1 (1.0)	1 (1.5)	2 (0.9)	3 (1.0)	2 (2.5)	0 (0)	1 (2.0)	1 (1.9)	2 (1.7)	3 (1.7)
**amblyopia**	1 (0.8)	0 (0)	0 (0)	0 (0)	1 (0.4)	1 (0.3)	1 (1.3)	0 (0)	0 (0)	0 (0)	1 (0.8)	1 (0.6)
**Other cause**	1 (0.8)	1 (1.5)	0 (0)	1 (1.5)	1 (0.4)	2 (0.7)	1 (1.3)	1 (2.3)	0 (0)	1 (1.9)	1 (0.8)	2 (1.2)
**Undetermined**	0 (0)	1 (1.5)	1 (1.0)	0 (0)	2 (0.9)	2 (0.7)	0 (0)	1 (2.3)	1 (2.0)	0 (0)	2 (1.7)	2 (1.2)
**Total**	120 (100.0)	67 (100.0)	104 (100.0)	66 (100.0)	225 (100.0)	291 (100.0)	79 (100.0)	44 (100.0)	49 (100.0)	52 (100.0)	120 (100.0)	172 (100.0)

## Discussion

This current multiethnic study provided vital epidemiological data for accurate comparison of three ethnic differences in the prevalence, surgical coverage, barriers and visual acuity outcomes of cataract surgery in adults aged ≥50 years in rural areas of southwest China.

A striking finding of this study is that people of different ethnicity have different cataract surgery uptake and visual outcomes, even if they reside in the same environment. One of the surprised things about our study is that CSC was the lowest in Han ethnicity among three ethnicities. The prevalence of cataract surgery in Han ethnicity was not low, but Han ethnicity was particularly affected by severe visual impairment or blindness because of unoperated cataract, as reflected in a greater total cataract social burden. To make matters worse, we found Han ethnicity was more likely to be associated with a poor outcome than Yi and Bai ethnicity. The prevalence of cataract surgery and cataract social burden in Bai ethnicity was the lowest, but CSC was not the lowest among three ethnics. CSC and better BCVA outcomes significantly differentially favored Yi ethnicity. The ethnic disparity in cataract social burden reflects the ethnic disparity in the prevalence of cataract-related severe visual impairment or blindness. The ethnic disparity in CSC reflects the ethnic disparity in the degree of cataract surgical services to meet the needs of people. Ethnic covers a number of factors including genetics, history, culture, language, religion or behavior. These factors could not be captured by current study. Since Chinese government’s preference policies, ethnic minorities got more opportunities for cataract operations compared with Han ethnicity. The sixth national census data show that Yunnan Han population accounts for 66.63% of the total population [26]. One of the pressing problems confronting us today is that large Han population increased the difficulty of prevention of blindness. Free cataract surgery is a benefiting people’s measure in China, but it only targets the poor, and is not the best way to solve the problem of cataract blindness. It is imperative to establish a new type of cooperative medical system in rural areas. Both the previous new rural cooperative medical insurance and the integrated urban and rural residents’ basic medical insurance have significantly reduced the proportion of farmers paying for cataract surgery. There is also a more preferential medical bill settlement policy for poor farmers. Eye care services can be made affordable by both the government and farmer.

Population-based studies have reported a wide range of prevalence of cataract surgery across different ethnic groups in different countries. For example, the prevalence of cataract surgery was reported to be 3.9% in Sri Lanka [4], 6.28% in Brazil [27], 7.0% in Nepal [28], 17.6% in India [29]. The prevalence of cataract surgery of 6.0% as found among the multiethnic people in this study was considerably higher than that found earlier in China [9, 10, 14–17]. The result indicates a striking increase in the prevalence of cataract surgery in rural China in recent years. The reasons may be as follows: First, the Chinese government attaches importance to the prevention of blindness in ethnic minority areas and has given preferential policies, financial subsidies and technical support. Second, with the rapid development of local economy, sustained effort on combating cataract blindness is performed in Yunnan in recent years. Furthermore, more and more non-governmental organizations are involved in the prevention and treatment of cataract blindness.

In this study, the CSC of the multiethnic population was 53.3%, which is at a medium level compared with previous studies in China [9, 10, 14–17] or other developing countries [4–8, 27–31]. For whatever reason, nearly half of Yunnan people with bilateral visual impairment or blindness because of cataract did not receive cataract surgery. The great social burden of cataract-related severe visual impairment or blindness of 7.9% as found among the multiethnic people in the study was remarkably higher than that found in the China Nine-Province Survey (3.83%) [17] and Beijing (3.48%) [16]. In this study, the social burden of cataract -related severe visual impairment or blindness was used as an index to estimate the severity of cataract. In the actual calculation, due to the lack of preoperative visual acuity data, we use the same method of the above two studies to make the necessary assumptions when identifying the cataract severe visual impairment or blind who have undergone cataract surgery. This is a fact that the magnitude of cataract blindness problem is high and the demand for service is obvious in rural Yunnan. This could be explained by multiple reasons. First of all, Yunnan is located in the Yunnan-Guizhou Plateau at high altitude and low latitude, with long sunshine time and high ultraviolet intensity. Moreover, farmers were exposed to high-intensity plateau ultraviolet radiation, working outdoors with low degree of agricultural mechanization and automation. Last but not least, other factors such as hypoxia, vitamin and micronutrient deficiencies may also play a role.

With regard to other risk factors, our study confirms the association of increased age, lower educational level, diabetes history, and no alcohol intake with higher prevalence of cataract surgery. As shown in Table 4 and Table 6, the Han ethnicity, the elderly and those with little or no education were particularly affected by a greater cataract social burden. Although with higher prevalence of cataract surgery, the elderly were particularly affected by severe visual impairment or blindness because of both post-operative and unoperated cataract. However, CSC significantly differentially favored younger people. The rapidly ageing population in China may be one of the reasons for the higher burden of visual impairment or blindness due to cataract. Although there was no significant association between gender and CSC, the overall risk of cataract-related severe impairment or blindness was higher in women, which was reflected in a larger total burden, as reflected in a greater total burden, as shown in Table 4. The barrier to surgery was lack of awareness and knowledge, cost, and fear.

Our findings indicate the main self-reported barriers to cataract surgery in multiethnic population were lack of awareness and knowledge, cost, and fear, similar to other previous studies [2–10]. First, knowledge and awareness was the main barrier for cataract surgery uptake. Most cataract blind people did not know what cataracts are and did not know that cataracts can be cured before their eyes were examined by us. They would not take the initiative to seek help from eye doctors, did not know how to get treatment and could not get timely, convenient medical treatment. Public health education should start at a young age. Second, cost was the main barrier for unwillingness to pay for cataract surgery. In recent years, the continued development of cataract blindness prevention programs has been popular in China. These projects address the massive backlog of cataract blindness. Most post-operative subjects in this study received free surgery. New cataract patients are determined to wait for the next free cataract operations and are very reluctant to undergo any form of paid surgery. However, no government, organization, or individual can afford to provide free cataract services permanently. Government should use limited funds make the treatment more cataract patients with severe visual impairment or blindness. Some cataract patients without severe visual impairment or blindness may be told to wait until the cataract gets mature. Third, fear was due to inadequate knowledge and awareness, and low quality of surgical outcome. We need to consider education, cost and quality of surgery to find a long-lasting and effective solution to improve cataract surgery uptake.

A good vision outcome (VA ≥ 20/63) was found in 44.5% of postoperative eyes with PVA and in 67.2% with BCVA in multiethnic population. According to the standards of World Health Organization [32], visual acuity outcomes of cataract surgery in multiethnic population in the study were generally poor except for the Yi people. Numerous factors come into play as to postoperative visual impairment in multiethnic population, including aphakia, Han ethnicity, earlier surgery year, illiteracy, and lower level surgical setting. The survey highlighted important aphakia associations with unsatisfactory cataract surgical outcomes. Visual outcome was better with IOL implantation than without it, as has been observed in many other studies [2, 7–9, 17–19, 29, 30]. Aphakic is more prone to surgical complications, and it is more difficult to obtain satisfactory refractive correction. The proportion of IOL implantation in multiethnic population was 86.7%. The proportion is similar to other economically developed areas, such as Liwan (87.8%) and Hong Kong (86.9%), is much higher than that in Tibet (40%) [9], Kunming (64.8%) [10], Shunyi (39.7%) [14], Doumen (5.9%) [15], Harbin (61.8%) [18], and the China Nine-Province Survey (78.4%) [17], and is much lower than those in Beijing (92.5%) [16]. The proportion of IOL implantation associated with ethnicity and year of surgery in our study. Yi ethnicity has the highest implantation rate, followed by Han ethnicity and Bai ethnicity. The visual outcomes of the eyes operated on after 2000 tended to be better than that of those operated on during or before 2000, due to improved surgical technique and higher proportion of pseudophakic. Illiteracies tended to be relatively poor visual outcomes with untreated surgical complications or concomitant diseases. Cataract surgeries performed on subjects in lower level surgical settings were more likely to be associated with a poor outcome than those in provincial or municipal hospitals, and there was a higher likelihood of free surgery in rural areas. There is a continued need for the advanced technology and trained surgeons to reduce surgical complications.

This study underscores the importance of retinal disorders, refractive error or uncorrected aphakia, PCO, and glaucoma as main causes of postoperative visual impairment in multiethnic population, similar to the findings in Liwan [19] and Hong Kong [20]. Some retinal diseases and glaucoma may exist before surgery, the other one may be gradually emerged with age after surgery, and others may be surgical complications. The tractable causes of impairment and blindness, such as refractive error, PCO, and pterygium, accounted for 50.2% of cataract-operated eyes with visual impairment in the study. The cause of refractive error is aphakic, excessive surgical incision, deviation of intraocular lens power measurement, and absence of optometry. The high prevalence of PCO is alarming due to the fact that there are very few Nd:YAG laser facilities outside tertiary hospitals in Yunnan. These findings emphasize the importance of strengthening preoperative screening, maintaining quality of surgery with IOL, postoperative follow-up, and providing corrective glasses if needed when increasing the number of surgeries.

The principal strengths of the study include multiethnic participants, a large sample size, and reasonable response rates. There were some limitations in this study. Firstly, the relatively low response rate of the youngest group (67.4%) and male (65.9%) are responsible for the 20% non-response rate, which was may lead to an overestimation of the true burden of cataract-related severe visual impairment and blindness. Those absent younger men may be healthier, who were working in urban cities. Secondly, when analyzing risk factors, the ability to assess causality is limited by cross-sectional design.

## Conclusions

Ethnicity was significantly associated with surgical uptake and visual outcomes of cataract surgery in a multiethnic adult population in rural China, with Han ethnicity having a lower CSC and relatively poor visual outcomes of cataract surgery compared with ethnic minorities. Knowledge and awareness, cost, fear barriers in this population impede access to surgery. Further effort to remove these barriers and provide sight restoration is warranted among multiethnic individuals living in rural south-western China. The sound data will be used during the prioritization of resources and planning of eye care programs in the region.

## Data Availability

The datasets used and/or analysed during the current study are available from the corresponding author on reasonable request.

## Abbreviations

WHO: World Health Organization
CSR: cataract surgical rate
CSC: Cataract surgical coverage
YMES: Yunnan Minority Eye Studies
PVA: Presenting visual acuity
BCVA: best corrected visual acuity
CIs: Confidence intervals
OR: odds ratios
PCO: posterior capsule opacification
IOL: intraocular len
